# Editorial: Fractures and deformities of the lower extremity in children and adolescents: etiology, diagnosis and treatment

**DOI:** 10.3389/fped.2024.1493293

**Published:** 2024-09-25

**Authors:** Shunyou Chen, Xin Tang, Federico Canavese

**Affiliations:** ^1^ Department of Pediatric Orthopedics, Fuzhou Second Hospital, Fuzhou, Fujian, China; ^2^ Department of Pediatric Orthopedics, Wuhan Union Hospital, Tongji Medical College, Huazhong University of Science and Technology, Wuhan, China; ^3^ Orthopedic and Traumatology Department, IRCCS Istituto Giannina Gaslini, Genoa, Italy; ^4^ Department of Integrated Surgical and Diagnostic Sciences (DISC), University of Genova, Genoa, Italy

**Keywords:** etiology, diagnosis and treatment fracture, deformity, lower extremity, children, treatment, outcome

**Editorial on the Research Topic**
Fractures and deformities of the lower extremity in children and adolescents: etiology, diagnosis and treatment

Orthopedic and trauma conditions in children can have a significant impact on lifestyle and mobility due to the rapidly growing skeletal structure of the patient, despite the extremely favorable healing potential of children. Pediatric orthopedic disorders and fractures present, are treated, and progress differently than those of adults and require special attention. Children are therefore a special group of patients with very specific and highly variable orthopaedic problems that require highly specialized orthopaedic care.

The anatomical characteristics of the pediatric bone are responsible for the peculiarities of pediatric orthopedic disorders and fractures, which require specific diagnostic methods and treatment options. Knowledge of the anatomy and characteristics of bone growth, as well as the specific dynamics in this age group, is essential for the treatment of such disorders and injuries. In particular, children are often treated as small adults, sometimes leading to inaccurate clinical assessment, misinterpretation of radiographic findings, inappropriate treatment selection and follow-up. Therefore, it is imperative that the pediatric orthopaedic surgeon managing patients with fractures have the knowledge and skills to competently apply all available techniques for proper management.

There is still much debate regarding the optimal management of pediatric traumatic and non-traumatic disorders, as the anatomic regions of interest vary between age groups. Although there have been tremendous advances in both non-operative and operative treatment techniques for pediatric traumatic and non-traumatic disorders in recent decades, current treatment strategies have relatively limited scientific evidence. In particular, traditional non-operative treatment guidelines have been challenged by surgical treatment. However, it is unclear whether this translates into improved outcomes. As a result, there are numerous techniques available, and the most effective technique for some pediatric traumatic and non-traumatic conditions has yet to be determined.

The field of pediatric orthopaedics and traumatology is approaching its adult counterpart in terms of increasing hyperspecialization, as reflected in the number and quality of scientific publications and the emergence of subspecialty journals. In such a diverse and rapidly changing environment, the aim of the special issue “*Fractures and Deformities of the Lower Extremity in Children and Adolescents: Etiology, Diagnosis and Treatment*”, co-edited by Xin Tang, hunyou Chen and Federico Canavese, was to collect scientific articles reporting the results of different treatment modalities of a wide spectrum of pediatric traumatic and non-traumatic lower extremity pathologies, of high scientific quality, representative of different academic and medical settings, and covering both clinical aspects and innovations in pediatric orthopaedics and trauma surgery. The different original articles were carefully analyzed by 2 to 4 reviewers and the 3 guest editors.

The special issue contains a total of 21 articles, of which 9 are trauma related Zhi et al., Ding et al., Miao et al., Li et al., Hu et al., Liu et al., Qiao et al., including one systematic review Li et al. and one narrative review Li et al., and 12 are non-trauma related Fang et al., Xie et al., Wang et al., Hong et al., Chen et al., Ren et al., Verdoni et al., Lu et al., Cuevas-Martínez et al., Wu et al., Li et al., Lin et al.

The original articles focusing on lower extremity trauma examined the epidemiology of anterior cruciate ligament and risk factors for concomitant meniscal tears Zhi et al., changes in serum vitamin C levels in children with limb fractures Ding et al., lower extremity alignment (LEA) in children with recurrent patellar dislocation Miao et al., the management of unstable subtrochanteric pediatric femoral fractures Li et al., the outcome of reimplantation of extruded bone segments in open lower limb fractures Hu et al., the surgical treatment of distal tibial diaphyseal-metaphyseal junction fractures Liu et al., and the outcome of diaphyseal femoral fractures in children aged 2–6 years. The systematic review compared the outcome of submuscular plating and elastic stable intramedullary nailing for diaphyseal femoral fractures Li et al., while the narrative review reported on arthroscopic fixation techniques for tibial eminence fractures.

Among the articles dealing with orthopedic pathologies, two evaluated the results of the Kidner procedure Fang et al. and arthroeresis for flexible flatfoot Xie et al.; four dealt with various aspects of developmental dysplasia of the hip, including the cartilage of the acetabulum Wang et al., Hong et al., the individual characteristics that may influence ultrasound examination Chen et al., and the effect of hip dysplasia on acetabular development Ren et al.; two dealt with lower limb deformities, including bilateral lower limb lengthening for achondroplasia Verdoni et al. and the use of interlocking nail fixation in the treatment of lower limb deformities Lu et al.; one reported on the impact of functional hallux limitus on quality of life Cuevas-Martínez et al.; one evaluated the radiographic appearance of congenital thumb duplication Wu et al.; one focused on the outcome of proximal femoral bone cysts Li et al.; and one assessed the clinical reliability and validity of a video-based markerless gait assessment method Lin et al.

The special issue should be of interest not only to pediatric orthopaedic surgeons, but also to adult orthopaedic surgeons interested in the latest news and trends in the rapidly evolving field of pediatric orthopaedic and trauma surgery.

The Guest Editors would like to express their gratitude to Frontiers for entrusting them with this challenging and rewarding task, and to all the authors whose research contributed to this special issue, without whose efforts nothing would have been possible.

